# Antimicrobial stewardship to reduce overtreatment of asymptomatic bacteriuria in critical access hospitals: measuring a quality improvement intervention

**DOI:** 10.1017/ice.2024.171

**Published:** 2025-02

**Authors:** Claire E. Ciarkowski, Hannah N. Imlay, Chloe Bryson-Cahn, Jeannie D. Chan, Whitney Hartlage, Adam L. Hersh, John B. Lynch, Natalia Martinez-Paz, Emily S. Spivak, Hannah Hardin, Andrea T. White, Chaorong Wu, Valerie M. Vaughn, Zahra Kassamali Escobar

**Affiliations:** 1 Division of General Internal Medicine, Department of Internal Medicine, University of Utah, Salt Lake City, UT, USA; 2 Division of Infectious Diseases, Department of Internal Medicine, University of Utah, Salt Lake City, UT, USA; 3 Veteran’s Affairs Salt Lake City Health Care System, Salt Lake City, UT, USA; 4 Center for Stewardship in Medicine, University of Washington, Seattle, WA, USA; 5 Division of Allergy and Infectious Diseases, University of Washington School of Medicine, Seattle, WA, USA; 6 School of Pharmacy, University of Washington, Seattle, WA, USA; 7 Division of Infectious Diseases, Department of Pediatrics, University of Utah, Salt Lake City, UT, USA; 8 Division of Epidemiology, Department of Internal Medicine, University of Utah, Salt Lake City, UT, USA

## Abstract

**Background:**

Asymptomatic bacteriuria (ASB) treatment is a common form of antibiotic overuse and diagnostic error. Antibiotic stewardship using the inappropriate diagnosis of urinary tract infection (ID-UTI) measure has reduced ASB treatment in diverse hospitals. However, critical access hospitals (CAHs) have differing resources that could impede stewardship. We aimed to determine if stewardship including the ID-UTI measure could reduce ASB treatment in CAHs.

**Methods:**

From October 2022 to July 2023, ten CAHs participated in an Intensive Quality Improvement Cohort (IQIC) program including 3 interventions to reduce ASB treatment: 1) learning labs (ie, didactics with shared learning), 2) mentoring, and 3) data-driven performance reports including hospital peer comparison based on the ID-UTI measure. To assess effectiveness of the IQIC program, change in the ID-UTI measure (ie, percentage of patients treated for a UTI who had ASB) was compared to two non-equivalent control outcomes (antibiotic duration and unjustified fluoroquinolone use).

**Results:**

Ten CAHs abstracted a total of 608 positive urine culture cases. Over the cohort period, the percentage of patients treated for a UTI who had ASB declined (aOR per month = 0.935, 95% CI: 0.873, 1.001, *P* = 0.055) from 28.4% (range across hospitals, 0%-63%) in the first to 18.6% (range, 0%-33%) in the final month. In contrast, antibiotic duration and unjustified fluoroquinolone use were unchanged (*P* = 0.768 and 0.567, respectively).

**Conclusions:**

The IQIC intervention, including learning labs, mentoring, and performance reports using the ID-UTI measure, was associated with a non-significant decrease in treatment of ASB, while control outcomes (duration and unjustified fluoroquinolone use) did not change.

## Introduction

Antibiotic overuse leads to adverse events, excess costs, and antibiotic resistance.^1^ Misdiagnosis and treatment of asymptomatic bacteriuria (ASB) are major contributors to antibiotic overuse and patient harm.^2^ Unlike urinary tract infections (UTI), antibiotic treatment of ASB does not improve outcomes, with few exceptions.^2,3^ Despite this, treatment of ASB remains common practice, with treatment rates as high as 80%.^1,4,5^ While interventions focusing on misdiagnosis of ASB as UTI have been successful, it is unclear whether similar strategies would be successful in hospitals with fewer resources.

One in five US adults lives in a rural area and receives care from rural or CAHs (ie, rural hospitals with <25 inpatient beds and located >35 miles from another hospital).^6–8^ Rural and CAHs typically have less infrastructure and resources for quality improvement (QI) which could impact antibiotic stewardship efforts.^9^ For example, rural hospitals in Michigan have more antibiotic overuse at discharge than non-rural hospitals.^10^ While antimicrobial stewardship programs are now required in all hospitals, resources (eg, dedicated time, information technology, and specialist expertise) for these programs vary greatly across hospitals.^11^ Prior quality and research efforts for antibiotic stewardship have primarily focused on larger hospitals; therefore, it is not well understood how to implement stewardship in CAHs.

To address the gap in stewardship for CAHs, the University of Washington Center for Stewardship in Medicine (UW CSiM) created a stewardship collaborative with CAHs to reduce ASB treatment (www.uwcsim.org). UW CSiM found overtreatment of ASB was common in CAHs, especially in their emergency departments (EDs).^12^ For example, up to 75% of cases identified as ASB in CAHs are treated with antibiotics.^12^ CSiM’s initial multipronged pilot QI program with 19 CAHs saw high engagement (eg, 95% of sites completed their plan/do/study/act cycle), but hospitals reported high-quality data as a primary barrier to improvement.^13^ Thus, for this cohort, we incorporated ASB measurement and hospital peer comparison using a National Quality Forum (NQF)-endorsed patient safety measure that defines inappropriate diagnosis of UTI (ID-UTI measure) and has been shown to significantly reduce ASB treatment in non-CAHs.^14,15^

## Methods

### Design, setting, and study population

This multisite QI study included CAHs that had already participated in a 1 year intensive quality improvement cohort (IQIC) focused on antimicrobial stewardship of UTIs in 2021 through UW CSiM.^13^ The UW CSiM pilot occurred as part of a QI initiative from the Office of Rural Health State Flex programs in Arizona, Idaho, Oregon, Utah, and Washington. This first cohort included 19 hospitals which were recruited by UW-CSiM and Medicare Rural Hospital Program coordinators in each state, and participation was voluntary. This collaborative partnership and participation in the UW-CSiM pilot program was financially supported through the Medicare Flex program and not the individual hospitals.^16^ The first-year pilot program combined education in UTI and QI (goal setting and process mapping), mentoring to identify their current processes related to urine culturing and UTI treatment (eg, how urine cultures were obtained), and non-standard data collection (not the ID-UTI measure) to address ASB.

For this study, we recruited hospitals that had participated in the CSiM pilot for an additional year of ASB QI work including data collection using the ID-UTI measure.

### Intervention

Hospitals that agreed to participate underwent 3 longitudinal interventions aimed at reducing treatment of ASB: 1) learning labs (ie, didactics with shared learning), 2) one-on-one mentoring, and 3) data-driven hospital performance reports using the ID-UTI measure. Each hospital identified at least one stewardship champion to attend educational and mentoring sessions and to submit local, de-identified data using the ID-UTI measure. At the beginning of the year, each participating hospital identified an ASB-related goal and a QI intervention (eg, orderset hygiene, reflex testing) to achieve that goal.

First, monthly group learning labs were held virtually and included a range of topics including diagnostic stewardship, challenging populations (eg, patients with altered mental status), and social-behavioral impacts on treatment (see appendix for syllabus). Learning labs included a short didactic presentation and facilitated discussion with interactive polling questions and time for participating hospitals to share progress or barriers. Second, a UW stewardship pharmacist (Z.K.E.) and either an Internal Medicine Hospitalist (C.C.) or an Infectious Disease physician (H.I.) from the University of Utah held quarterly individual 30-minute virtual mentoring sessions with stewardship champions from each participating CAH. Mentoring sessions were individualized to hospital needs (ie, their identified goals/QI project) to address barriers. Finally, hospitals received hospital performance reports every two months containing hospital-specific ASB treatment data based on the ID-UTI measure. Performance reports included change over time in the hospitals ID-UTI measure as well as benchmarked data compared to other participating CAHs and national estimates (see appendix for example). We did not dictate how ID-UTI data were to be used, though mentoring sessions included strategies for stewardship champions to provide feedback from hospital report cards to clinicians and hospital leadership (eg, didactic sessions). Customized suggestions included ideas on who to engage, how to share data at staff meetings, how to praise top performers, as well as individual feedback (eg, handshake stewardship and “dear doctor” letters). While labs, mentoring, and data reports discussed UTI and ASB-related antibiotic use broadly, the main focus was reduction of ASB detection and treatment.

### ID-UTI data collection

Over a 10-month period (October 2022 to July 2023), each CAH submitted a convenience sample of cases with bacteriuria (including both UTI and ASB) treated with antibiotics. Cases included inpatients and/or in those seen in the ED. Consistent with the ID-UTI measure, CAHs were instructed to exclude patients if they were < 18 years old, had a patient-directed discharge, were admitted on hospice, were pregnant, had a history of spinal cord injury, received an antibiotic prescription for >14 days (proxy for identifying a complicated infection), or had a concomitant non-UTI indication for antibiotic therapy. Bacteriuria was identified by urine cultures flagged as “abnormal” by each hospital’s clinical laboratory and EMR; no specific colony threshold was utilized. UTI was defined, per the ID-UTI measure, by the presence of any of the following symptoms documented in the medical record: urgency, rigors, frequency, dysuria, suprapubic pain or tenderness, acute hematuria, costovertebral or flank pain or tenderness, documentation of pyelonephritis, fever >38C, or new onset mental status changes with systemic signs of infection.^2^ ASB was defined as treated bacteriuria in the absence of symptoms of UTI. Chart abstraction was performed by stewardship champions or a surrogate (all non-physicians) with deidentified data submitted via an electronic REDCap tool. Champions were provided a data dictionary and manual, but no specific training on data collection was provided. To ensure appropriate selection of patients, if an abstractor input a chart meeting the ID-UTI exclusion criterion, REDCap would flag the abstractor to stop inputting data. We set a monthly data collection goal of 5-6 cases per CAH which, over 10 months, would equate to 59 cases, a number previously shown to achieve “high” reliability of 0.8 for assessing ASB treatment.^14^ Notably, moderate reliability (0.6) required a minimum of 22 cases to be submitted and good reliability (0.7) required a minimum of 35 cases.^14^

### Study outcomes

Our primary aim was to determine change over the intervention period in inappropriate diagnosis and treatment of ASB assessed using the ID-UTI measure which quantifies the percentage of treated ASB cases relative to all cases of treated bacteriuria (ASB + UTI).^14^ The measure can be improved by either decreasing treatment of ASB or decreasing urine cultures obtained in asymptomatic patients. To reduce confounding by time, we compared change in the ID-UTI measure to change in two non-equivalent dependent variables (ie, non-equivalent concurrent controls)—total antibiotic duration and percentage of cases with potentially unjustified fluoroquinolone use.^17^ While data on antibiotic duration and fluoroquinolone use were provided in the bimonthly performance reports, duration and fluoroquinolones were not used to benchmark hospitals nor were they the focus of the 10-month curriculum. Potentially justified fluoroquinolone use was defined as fluoroquinolone use in patients with UTI who had a fever, documented pyelonephritis, or 2 or more systemic inflammatory response syndrome (SIRS) criteria. Fluoroquinolone use in patients not meeting these criteria was considered “potentially unjustified.” Antibiotic duration included both UTI and ASB cases.

### Analyses

Patient characteristics and antibiotic treatment were characterized using descriptive statistics. To determine change over time for the primary outcome (the ID-UTI measure) and concurrent control outcomes (duration of antibiotics and potentially unjustified fluoroquinolone use), we employed generalized linear mixed models with logistic or identity link functions as appropriate. Models controlled for clustering by hospital with results presented as adjusted odds ratios (aORs) or adjusted regression coefficients per month (ie, comparing each month to the prior month). Consistent with the ID-UTI measure,^14^ we did not adjust for individual patient characteristics. All analyses were performed using R Statistical Software (v 4.3.1; R Core Team 2023) with a *P*-value <0.05 considered significant. This project was reviewed by the University of Utah IRB and received “exempt” status. We followed SQUIRE reporting guidelines for QI (appendix).^18^

## Results

Of the 19 CSiM pilot hospitals, 14 CAHs initially volunteered. However, two dropped out prior to program initiation due to loss of their stewardship champion and two submitted data for 1 month only and were excluded from final analysis. The 10 included CAHs were located in the Pacific or Mountain West region of the United States. Sixty percent (6/10) of participating stewardship champions were pharmacists (Table 1).

**Table 1.** Characteristics of participating critical access hospitals

Hospital Characteristic	Critical Access Hospitals (N = 10)
**Location** Arizona Idaho Oregon Utah Washington	1 3 3 1 2
**Role of Antimicrobial Stewardship Champion** Compliance Infection prevention Pharmacist Nurse	1 1 6 2
**Time Spent Dedicated to Stewardship** < 25% 25–49% > = 50% Unknown	5 4 0 1
**Number of Educational Sessions Attended out of 10,** Median (IQR)	9 (9,10)
**Number of Mentoring Sessions Attended out of 3,** Median (IQR)	3 (3,3)
**Hospitals’ Stated Quality Improvement Goals** Educate providers Reduce treatment rate of ASB Improve antibiotic treatment duration Change UA reflex criteria Reduce routine ordering of UAs in surgical patients Evaluate antibiotic appropriateness Track prescribing data	4 3 2 1 1 2 2

Abbreviations: IQR, iInterquartile range; ASB, asymptomatic bacteriuria; UA, urinalysis.

**Figure 1.**
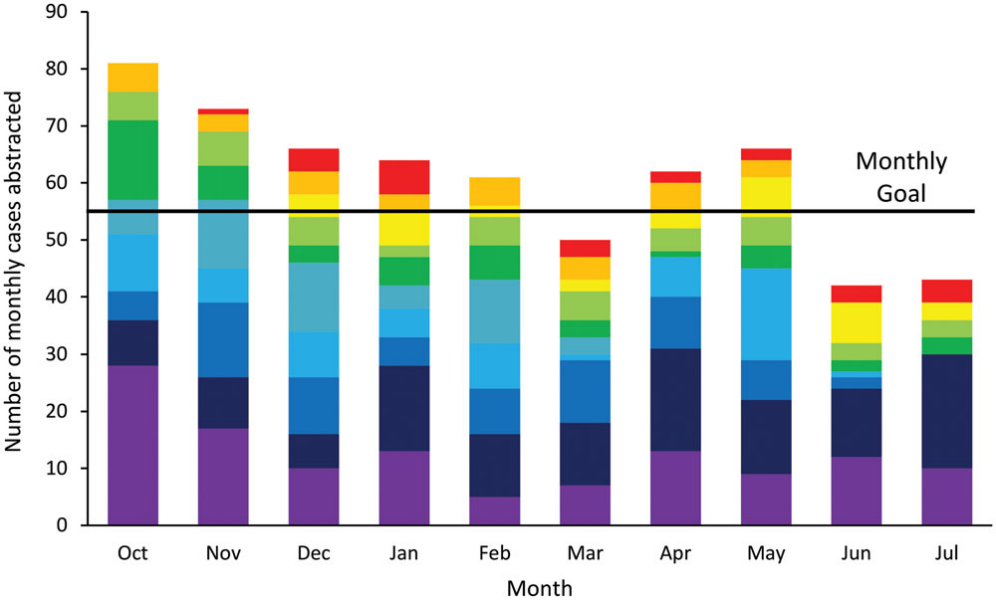
Number of cases submitted over time, hospitals combined. Figure Legend: Bars reflect total number of cases submitted by each hospital, colors represent individual critical access hospitals.

All sites (10/10) attended at least 50% of the virtual monthly learning labs and eight sites completed all quarterly mentoring meetings. Across all 10 CAHs, 608 positive urine culture cases were abstracted. Of the 10 included hospitals, 4/10 (40%) abstracted 59 or more cases (goal); 3/10 (30%) abstracted 35–58 cases, and 3/10 (30%) abstracted 22-34 cases to achieve the pre-specified measure reliability targets of 0.80 (high), 0.70 (good), and 0.60 (moderate), respectively. The median abstraction time per case was 11 minutes (IQR 7.0 to 18.0). The number of cases submitted by hospitals decreased over time (Relative Risk [RR] = 0.966, 95% CI: 0.938, 0.996, *P* = 0.02) [Figure 1].

Seventy-five percent (455/608) of patients with bacteriuria were female, and the median age was 69.0 (IQR 45, 79) years (Table 2). Most patients (76% [463/608]) were seen in the ED and discharged. Almost all (97.5% [593/608]) urine cultures were collected in the emergency room; only 2.5% (15/608) were collected during hospitalization. Diabetes was the most commonly documented comorbidity, present among 25% (151/608) of patients. Patients requiring chronic catheterization (either indwelling or chronic straight catheterization) accounted for 9% (54/608) of cases. The most common organisms identified in the urine culture were *Escherichia coli* (66.1% [402/608]) and Klebsiella species (11.5% [70/608]). Approximately half of patients (53.8% [327/608]) received an intravenous (IV) antibiotic, most commonly ceftriaxone (86% [284/327]). Almost all patients (87.3% [531/608]) received an oral antibiotic on discharge—the most common were cephalexin (25% [133/531]), nitrofurantoin (16.2% [86/531]), and trimethoprim-sulfamethoxazole (13.7% [73/531]). A total of 106 patients (17% [106/608]) received a fluoroquinolone of which 61% (65/106) were classified as potentially unjustified. The median total duration of antibiotics for all patients was 8 days (IQR 6.0 to 10.0). Median (IQR) antibiotic duration for ASB was 7 (6, 9) days and for UTI was 8 (6, 10) days. Overall, 52.8% (321/608) of patients received greater than 7 days of antibiotics.

**Table 2.** Patient demographic, clinical, and treatment characteristics across all hospitals

Variable	All Hospitals n = 608 cases
Age, years; median (IQR)	69 (45,79)
Gender; Female, n (%)	450 (74%)
Race; n (%)	
White	393 (65%)
American Indian/Alaska Native ^a^	44 (7%)
Black	1 (<1%)
Unknown/Not Specified	170 (28%)
Ethnicity; n (%)	
Hispanic/LatinX	25 (4%)
Not Hispanic/LatinX	372 (61%)
Unknown/Not Specified	211 (35%)
Location (time of culture); n (%)	
ED, then discharged	463 (76%)
ED, then admitted/inpatient	145 (23%)
Culture reflexed from urinalysis; Yes, n (%)	549 (90%)
Comorbidities; n (%)	
None	343 (56%)
Diabetes	151 (25%)
Dementia	51 (8%)
Immune suppression (mild)	18 (3%)
Urologic comorbidities; n (%)	
Chronic catheterization (any)	54 (9%)
Neurogenic bladder/retention	34 (6%)
Primary urine culture organism, n (%)	
*Escherichia coli*	402 (66.1%)
*Klebsiella* species	70 (11.5%)
*Enterococcus* species	30 (4.9%)
*Proteus mirabilis*	23 (3.8%)
Other	112 (18.4%)
IV Antibiotics prescribed^b^; inpatient/ED, n (%)	327 (53.8%)
Ceftriaxone	284 (86.9%)
Piperacillin-tazobactam	17 (5.2%)
Ciprofloxacin	15 (4.6%)
Oral antibiotics prescribed^b^; inpatient/ED, n (%)	216 (35.5%)
Cephalexin	53 (24.5%)
Nitrofurantoin	47 (21.8%)
Trimethoprim-Sulfamethoxazole	28 (12.9%)
Oral antibiotics prescribed^b^; discharge, n (%)	531 (87.3%)
Cephalexin	133 (25.0%)
Nitrofurantoin	86 (16.2%)
Trimethoprim-Sulfamethoxazole	73 (13.7%)
Antibiotic treatment duration; days, median (IQR)	
Total duration	8 (6.0, 10.0)
Inpatient/ED duration	1 (1.0, 1.0)
Discharge duration	7 (5.0, 8.6)

Abbreviations: IQR, interquartile range; ED, emergency department.

a 89% of these patients were located in one critical access hospital.

b The three most commonly prescribed antibiotics in each category are listed.

**Figure 2.**
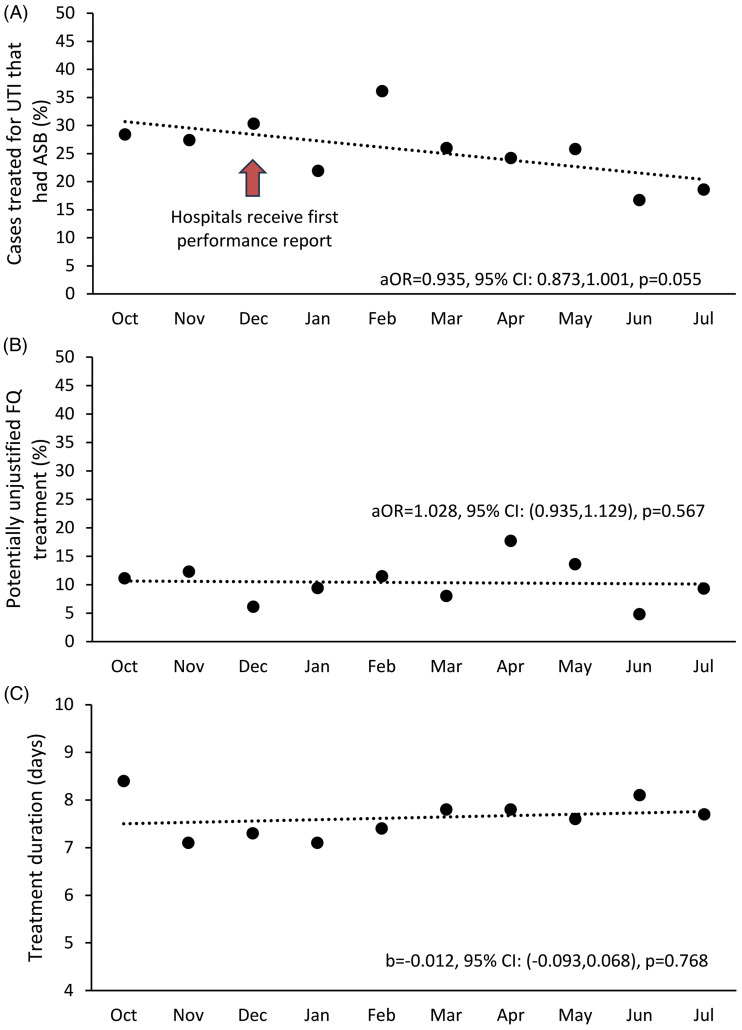
Inappropriate diagnosis of urinary tract infection, potentially unjustified fluoroquinolone use, and antibiotic treatment duration over time. Abbreviations: ASB, asymptomatic bacteriuria; UTI, urinary tract infection; FQ, fluoroquinolone. Across participating hospitals (n = 10), percentage of cases with ASB that were treated for UTI (panel A) decreased over time, while potentially unjustified fluoroquinolone treatment (panel B) and mean treatment duration for UTI and ASB (days; panel C) did not change. The arrow in panel A indicates when the first hospital feedback reports were distributed. Each dot represents the unadjusted hospital mean; the lines represent the logistic or identity link models as appropriate controlling for clustering by hospital.

During the study period the total treatment of ASB as a percentage of all treated bacteriuria, defined by the ID-UTI measure, was 26.2% (159/608). ASB treatment was 28.4% in October 2022 (first study mo) and decreased to 18.6% by the final month (July 2023), a reduction of 9.8% (95% CI: −5.4%, 25.0%) over the 10-month period (Figure 2, panel A). After adjusting for hospital clustering, the odds that a case treated for UTI was ASB non significantly decreased by 6.5% with each additional month (aOR = 0.935, 95% CI: 0.873, 1.001, *P* = 0.055). In contrast, after adjusting for clustering, there was no significant changes in antibiotic duration (unstandardized coefficient b = −0.012, 95% CI: −0.093, 0.068, [b can be interpreted as a 0.012 day decrease in antibiotic duration per month] p = 0.768) or the percentage of bacteriuric patients with potentially unjustified fluoroquinolone use (aOR = 1.028, 95% CI: 0.935, 1.129, *P* = 0.567) over time (Figure 2, panels B and C).

## Discussion

In this quasi-experimental QI study addressing ASB treatment across 10 CAHs, we found the IQIC intervention was associated with a non-statistically significant decline in the percentage of patients with ASB inappropriately treated for a UTI, suggesting the ID-UTI measure could potentially be used in CAHs to track antimicrobial stewardship of UTI and reduce overtreatment of ASB.

Although the reduction in ASB treatment was not statistically significant, the estimated effect size was fairly substantial and thus potentially clinically significant. We believe these findings likely represent true reduction in ASB treatment for the following reasons. First, our sample size was limited both in terms of participating hospitals and cases reviewed, and the period was somewhat short at 10 months. Second, the control variables (unjustified fluoroquinolone use and antibiotic duration) did not show a statistically significant, or clinically significant, change during this same period. While these results are promising, barriers should be noted. First, we lost 4 of the original 14 hospitals mostly due to stewardship turnover. Staffing and continuity of QI and stewardship leadership is a critical problem in CAHs. Second, data collection decreased over time. While most hospitals still collected enough cases to achieve “good” reliability, this decrease highlights the difficulty CAHs have in sustaining QI long term without dedicated resources. Future work over a longer period should be done to ensure that the decrease in overtreatment in ASB can be sustained.

Key differences about ASB in CAHs should be noted. First, most urine cultures were obtained and treated in the ED. As 76% of patients were discharged from the ED, antibiotic prescribing was often based on clinical suspicion and/or point of care urinalysis or dipstick rather than urine culture results. Interestingly, 56% of patients who received antibiotics did not have any documented comorbidities, making this CAH population less complicated than those seen in other studies of acute care hospitals or long-term care facilities.^19^ Despite lower rates of hospitalization and co-morbidities, the rate of ASB treatment in the CAHs participating in this study, 26.2%, was comparable to the 23.3% in the initial ID-UTI cohort (69 non-CAH in Michigan).^15^

CAHs, by definition, are small and remote. On the one hand, their smaller size and workforce allows for leanness in change management and QI. On the other hand, hospital staff, including antimicrobial stewardship champions who participated in this cohort, have multiple roles and responsibilities. The majority of stewardship champions were pharmacists who were able to devote less than 25% of their time to antimicrobial stewardship. CAH remoteness can limit access to resources and lead to discrepancies in equity of care. While all CAHs are small and remote, CAHs differ in their resources and populations served (eg, one participating hospital served primarily tribal populations). Specific challenges reported during the pilot year of the program including loss of stewardship champions, lack of dedicated stewardship resources, and difficulty with data collection due to limited capacity of EMRs. Despite ongoing challenges, participating CAHs in this second year remained highly motivated, and the stewardship champions continued to attend learning labs. During mentoring calls, CAHs reported that working in this multi-hospital collaborative and being able to compare their data and benchmarks as a group helped foster a sense of community and enhance learning opportunities.

Our study had several limitations. First, we were underpowered to detect a change over time in individual sites. Only 4 of 10 hospitals submitted the target 59 cases over the 10-month period due to low volumes of cases and competing priorities. Second, the ID-UTI measure requires manual data extraction which can increase the workload of stewardship champions. Given the variety of EMRs used and limited informatics infrastructure at these CAHs, an electronic alternative was not feasible. Third, the CAH stewardship champions participating in the program were a self-selected and institutionally supported group who might not be representative of all CAHs. Participating hospitals, though representative of CAHs demographically, may not be generalizable to all CAHs as they had already participated in the IQIC pilot year and had volunteered to continue participating to improve ASB. Fourth, as this study used a multi-modal approach to QI including use of the ID-UTI measure, education and mentoring, specific attributions to the reduction in overtreatment of ASB could not be assigned to each component. UW CSiM contributed resources for this multimodal approach which might not be generalizable to other CAHs not enrolled in the program. Fifth, the measure is based on both accurate assessment and documentation in the EMR and has been shown to overestimate UTI.^14^ Sixth, the use of convenience sampling may have led to selection bias. Seventh, the reliability thresholds used in this study were derived from a large cohort of hospitals and may not directly apply to CAHs. Lastly, we did not have a baseline period for comparison. Our study also has strengths. To our knowledge, it is the first to demonstrate use of a QI strategy including data collection and hospital benchmarking to reduce treatment of ASB in CAHs.^20^ CAHs often are unable to compare to peer institutions and thus have difficulty understanding their performance compared to very different populations usually included in antibiotic stewardship publications. Additionally, the use of two non-equivalent dependent variables is a methodologic strength which suggests improvements were not confounded by time.

In summary, a multipronged QI intervention including use of the ID-UTI measure appeared somewhat effective for stewardship champions to track and reduce treatment of ASB in CAHs. While barriers, including champion turnover and sustainability of data collection, may impact long-term sustainability; short-term, we found the ID-UTI measure, embedded in an environment of collaborative learning and hospital peer comparison can be implemented in resource-limited settings. By collecting, visualizing, and sharing data from rural patient populations who are often excluded from research and therefore data-driven policy decisions, we hope to promote equitable care for all patients.

## Supporting information

Ciarkowski et al. supplementary material Ciarkowski et al. supplementary material

## Supplementary material

The supplementary material for this article can be found at https://doi.org/10.1017/ice.2024.171
