# Supplementary material for: Antimicrobial stewardship to reduce overtreatment of asymptomatic bacteriuria in critical access hospitals: measuring a quality improvement intervention
Source: Infect Control Hosp Epidemiol. 2024 Nov 11;46(2):143–9. doi: 10.1017/ice.2024.171 (PMC11790331; doi:10.1017/ice.2024.171)
Supplement: Ciarkowski et al. supplementary material [file S0899823X24001715sup001.docx]

**Appendix**

Antimicrobial Stewardship to Reduce Overtreatment of Asymptomatic Bacteriuria in Critical Access Hospitals: Measuring a Quality Improvement Intervention

| SQUIRE Checklist | 1 |
| --- | --- |
| Bimonthly Performance Feedback Report - Example | 4 |
| Education Syllabus | 6 |

Revised Standards for Quality Improvement Reporting Excellence (SQUIRE 2.0) September 15, 2015

| **Text Section and Item**  **Name** | | **Section or Item Description** | |  |
| --- | --- | --- | --- | --- |
| **Title and Abstract** | |  | |  |
| **1. Title** | | Indicate that the manuscript concerns an initiative to improve healthcare (broadly defined to include the quality, safety, effectiveness, patient-centeredness, timeliness, cost,  efficiency, and equity of healthcare) | | Page 1 |
| **2. Abstract** | | 1. Provide adequate information to aid in searching and indexing 2. Summarize all key information from various sections of the text using the abstract format of the intended publication or a structured summary such as: background, local problem, methods, interventions, results, conclusions | | Page 2 |
| **Introduction** | | *Why did you start?* | |  |
| **3. Problem Description** | | Nature and significance of the local problem | | Page 3 |
| **4. Available knowledge** | | Summary of what is currently known about the problem, including relevant previous studies | | Page 3 |
| **5. Rationale** | | Informal or formal frameworks, models, concepts, and/or theories used to explain the problem, any reasons or  assumptions that were used to develop the intervention(s), and reasons why the intervention(s) was expected to work | | Page 3 |
| **6. Specific aims** | | Purpose of the project and of this report | | Page 3 |
| **Methods** | | *What did you do?* | |  |
| **7. Context** | | Contextual elements considered important at the outset of introducing the intervention(s) | | Page 3 |
| **8. Intervention(s)** | | 1. Description of the intervention(s) in sufficient detail that others could reproduce it 2. Specifics of the team involved in the work | | Page 4 |
| **9. Study of the Intervention(s)** | | 1. Approach chosen for assessing the impact of the intervention(s) 2. Approach used to establish whether the observed outcomes were due to the intervention(s) | | Page 4 |
| **10. Measures** | | 1. Measures chosen for studying processes and outcomes of the intervention(s), including rationale for choosing them, their operational definitions, and their validity and reliability 2. Description of the approach to the ongoing assessment of contextual elements that contributed to the success, failure, efficiency, and cost 3. Methods employed for assessing completeness and accuracy of data | | Page 5 |
| **11. Analysis** | | 1. Qualitative and quantitative methods used to draw inferences from the data 2. Methods for understanding variation within the data, including the effects of time as a variable | | Page 6 |
| **12. Ethical**  **Considerations** | | Ethical aspects of implementing and studying the intervention(s) and how they were addressed, including, but not limited to, formal ethics review and potential conflict(s) of interest | | Page 3 |
| **Results** | | *What did you find?* |  | |
| **13. Results** | | 1. Initial steps of the intervention(s) and their evolution over time (*e.g.*, time-line diagram, flow chart, or table), including modifications made to the intervention during the project 2. Details of the process measures and outcome 3. Contextual elements that interacted with the intervention(s) 4. Observed associations between outcomes, interventions, and relevant contextual elements 5. Unintended consequences such as unexpected benefits, problems, failures, or costs associated with the intervention(s). 6. Details about missing data | Page 6 &7 | |
| **Discussion** | | *What does it mean?* |  | |
| **14. Summary** | | 1. Key findings, including relevance to the rationale and specific aims 2. Particular strengths of the project | Page 7 | |
| **15. Interpretation** | | 1. Nature of the association between the intervention(s) and the outcomes 2. Comparison of results with findings from other publications 3. Impact of the project on people and systems 4. Reasons for any differences between observed and anticipated outcomes, including the influence of context 5. Costs and strategic trade-offs, including opportunity costs | Page 7&8 | |
| **16. Limitations** | | 1. Limits to the generalizability of the work 2. Factors that might have limited internal validity such as confounding, bias, or imprecision in the design, methods, measurement, or analysis 3. Efforts made to minimize and adjust for limitations | Page 9 | |
| **17. Conclusions** | | 1. Usefulness of the work 2. Sustainability 3. Potential for spread to other contexts 4. Implications for practice and for further study in the field 5. Suggested next steps | Page 9 | |
| **Other information** | |  |  | |
| **18. Funding** | | Sources of funding that supported this work. Role, if any, of the funding organization in the design, implementation,  interpretation, and reporting | Page 9 | |

**Bimonthly Performance Feedback Report –** *(Hypothetical Example)*

[Hospital Name] Asymptomatic Bacteriuria Report

[Month] 2022

Percent of Cases treated with Antibiotics that were Asymptomatic Bacteriuria (ASB)

Better

| Number of total cases (project goal 59): xx Number of cases this month (goal 6): xx | | | | | | | | |
| --- | --- | --- | --- | --- | --- | --- | --- | --- |
| Case IDs for the last 2 months’ Treated ASB Cases: xxx, xxx | | | | | | | | |
| **Table 1: Three most Common Antibiotics for Empiric and Discharge comparing UTI & ASB -** *Example* | | | | | | | | |
| **Empiric** | | | | | **Discharge** | | | |
| **UTI, n=x** | | **ASB, n=x** | | | **UTI, n=x** | | **ASB, n=x** | |
| e.g. Ceftriaxone, n=x (x%) | | e.g. Ceftriaxone, n=x (x%) | | | e.g. Cephalexin, n=x (x%) | | e.g. Cephalexin, n=x (x%) | |
| e.g. Cephalexin, n=x (x%) | | e.g. Cephalexin, n=x (x%) | | | e.g. Levofloxacin, n=x (x%) | | e.g. Amoxicillin-clavulanate, n=x (x%) | |
| e.g. Levofloxacin, n=x (x%) | | e.g. Amoxicillin-clavulanate, n=x (x%) | | | e.g. Cefdinir, n=x (x%) | |  | |
|  |  |  |  |  |  |  |  |  |
| **Table 2: Antibiotic Duration: Apr vs prior** | | | **UTI** | | | **ASB** | | |
| **Antibiotic Duration (days)** | | | **Prior, n=x** | **Apr, n=x** | | **Prior, n=x** | | **Apr, n=x** |
| Total Antibiotic Duration; median (IQR) | | | x (x,x) | x (x,x) | | x (x,x) | | x (x,x) |
| Inpatient duration; median (IQR) | | | x (x,x) | x (x,x) | | x (x,x) | | x (x,x) |
| Discharge duration; median (IQR) | | | x (x,x) | x (x,x) | | x (x,x) | | x (x,x) |
| Number of Cases Receiving >7 days; n (%) | | | x (x%) | x (x%) | | x (x%) | | x (x%) |
|  |  |  |  |  |  |  |  |  |

| **Table 3: Patient Characteristics: Apr vs prior n (%)** | **UTI** | | **ASB** | |
| --- | --- | --- | --- | --- |
| **Characteristics; n (%)** | **Prior, n=x** | **Apr, n=x** | **Prior, n=8** | **Apr, n=x** |
| Setting where culture obtained |  |  |  |  |
| ED, then admitted | x (x%) | x (x%) | x (x%) | x (x%) |
| ED, then discharged | x (x%) | x (x%) | x (x%) | x (x%) |
| Other | x (x%) | x (x%) | x (x%) | x (x%) |
| From reflex test | x (x%) | x (x%) | x (x%) | x (x%) |
| Men | x (x%) | x (x%) | x (x%) | x (x%) |
| Age >75 | x (x%) | x (x%) | x (x%) | x (x%) |
| Dementia | x (x%) | x (x%) | x (x%) | x (x%) |
| Admitted from SNF or LTAC | x (x%) | x (x%) | x (x%) | x (x%) |
| End stage renal disease | x (x%) | x (x%) | x (x%) | x (x%) |
| Immune suppression | x (x%) | x (x%) | x (x%) | x (x%) |
| Chronic catheter use | x (x%) | x (x%) | x (x%) | x (x%) |
| Chronic urinary retention | x (x%) | x (x%) | x (x%) | x (x%) |
| Had altered mental status with or without symptoms | x (x%) | x (x%) | x (x%) | x (x%) |
| Had altered mental status without signs of infection | x (x%) | x (x%) | x (x%) | x (x%) |
| Had >=2 SIRS Criteria on any day | x (x%) | x (x%) | x (x%) | x (x%) |

Action Items/Insight: Great job collecting cases! ___ seems to be a high performer in terms of avoiding antibiotic use for ASB, great work! Thanks for your hard work.

**IQIC ASB 201 Syllabus**

September 2022 – August 2023

| **Date** | **Learning Labs** | **Mentoring** |
| --- | --- | --- |
| September 9/28/22 | Kick off meeting – Introductions, Purpose, Goals Review of Data collection tool | How site will identify and record cases  AMS goal setting – continuing from previous year |
| October 10/26/22 | Getting down with the data |  |
| November 11/30/22 | Diagnostic Stewardship Debrief- what does it look like | How does your site collect urine cultures? |
| January  1/4/23 | Workshop – Urine culture collection Challenging cases: ASB with MDR pathogens Hospital Highlight |  |
| January 1/25/23 | Data validity and feedback Hospital Highlight |  |
| February 2/22/23 | Social-Behavior Impact on treatment  Data review so far | Check in on SMART goal and stewardship intervention(s) |
| March 3/22/23 | Challenging populations (CA-ASB) Hospital Highlight |  |
| April 4/26/23 | Challenging populations (AMS) | Check in on SMART goal and stewardship intervention(s) |
| May 5/24/23 | Data validity and feedback Introduce research template slide deck |  |
| June 6/28/23 | Stewardship, Data, Feasibility, Sustainability | No scheduled Check in |
| July 7/19/23 | 5 CAH give 10 min presentations |  |
| August 8/16/23 | 5 CAH give 10 min presentations |  |

All learning labs and mentoring sessions were held virtually over 1 hour and 30 minutes, respectively. In the learning lab, short didactics and facilitated discussions were presented by UW-CSiM and University of Utah faculty (MD/DO, PharmD) as well as guest faculty (PhD, MD/DO, PharmD). Each lab included dedicated time, at least 10 minutes, to allow stewardship champions to share their quality improvement progress and ask questions of both the faculty as well as their peers. Interactive polling was used frequently to help with engagement. Two labs were spent reviewing deidentified patient cases to address clinical quandaries and to gain consensus on how to approach them. In the final learning labs, each stewardship champion presented a “final presentation” of their own QI project and data. Learning labs were recorded and posted online for participating CAHs. Attendance was tracked and no CME/CPE was provided.
